# How Scientists View Vaccine Hesitancy

**DOI:** 10.3390/vaccines11071208

**Published:** 2023-07-06

**Authors:** Eric W. Welch, Timothy P. Johnson, Tipeng Chen, Jinghuan Ma, Shaika Islam, Lesley Forst Michalegko, Mattia Caldarulo, Ashlee Frandell

**Affiliations:** 1 Center for Science, Technology and Environmental Policy Studies, School of Public Affairs, Arizona State University, Phoenix, AZ 85004, USA; 2 Montpellier Advanced Knowledge Institute on Transitions, University of Montpellier, 34000 Montpellier, France; 3 Department of Public Policy, Management, and Analytics, University of Illinois at Chicago, Chicago, IL 60607, USA

**Keywords:** vaccine hesitancy, health crisis, science controversy, science communication, scientist roles, scientist opinions, risk, decision making

## Abstract

This paper examines possible causes, consequences, and potential solutions for addressing vaccine hesitancy in the United States, focusing on the perspectives of academic scientists. By examining the experiences of scientists, who are arguably a critical community in US society, we gain deeper insights into how they understand the complexities of vaccine hesitancy and whether their insights and opinions converge with or diverge from the current literature. We present findings from a national survey of a representative sample of academic scientists from the fields of biology and public health regarding vaccine hesitancy and related topics. Empirical analysis using descriptive, bivariate, and multivariate analyses covers multiple topics, including vaccine controversy, trust in science, causes of vaccine hesitancy, preferred policy and regulatory approaches, risk perceptions, and scientists’ ethics and perceived communication roles. The results highlight a diversity of opinions within the scientific community regarding how to improve science-society communication in regard to vaccines, including the need to be transparent and candid to the public about the risk of vaccines and their research.

## 1. Introduction

In times of crisis, societies often rely on science to navigate through the careful structuring of the problem and the development of solutions. The COVID-19 pandemic was no different. In response to the global pandemic, governments invested billions of dollars in scientific research, with the expectation that it would produce knowledge and technologies that would minimize the suffering and death associated with the disease. This was particularly true in the United States, where heavy public investments went into vaccine development [1], resulting in new vaccines and vaccine technologies that have had significant and far-reaching economic and social impacts [2,3].

Nevertheless, this science-society model does not appear to have worked as expected. COVID-19 vaccination levels were lower than expected during the pandemic, and they are continuing to fall. Public confidence in government, healthcare and science institutions has dropped [4,5], and substantial disagreement persists about the value that vaccines have had in stemming the pandemic, often along political divides [6]. Additionally, while investments in research continue to pour in [7], numerous states have enacted constraints on the government’s ability to set emergency health protection policies in the future [8]. We are left with many questions about why we did not do better and what can be done to improve.

A large amount of new retrospective investigation is underway to understand and learn from our collective experience. Most of that work relies on health policy experts who evaluate epidemiological data, policy data, or survey data gathered from the public [9,10,11,12,13,14,15]. Rarely do we directly hear the opinions of the science community on topics such as health risks, health policy responses, and public health behaviors. One exception is the recent interview of Dr. Anthony Fauci by the New York Times [16], which explored the sometimes-contradictory perspectives of a career scientist and science administrator who has been active at the forefront of the science-society interface for many years, and most recently known for communicating and brokering across science-policy boundaries on a daily basis during the COVID-19 pandemic [17]. Yet, because journalism necessarily selects representatives and filters their opinions to produce newsworthy stories, the diversity and complexity of opinions held by the broader science community remain largely invisible. To address rising public distrust of science and scientists, it is increasingly important to find new ways to enrich communication at the science–society interface. 

This paper advances our understanding of the diversity of scientists’ opinions related to vaccine hesitancy in the US. We ask three primary questions: What do academic scientists believe are the causes and consequences of vaccine hesitancy? What interventions does the science community think are best suited to minimize or reduce vaccine hesitancy? What roles do scientists have in addressing vaccine hesitancy? To answer these questions, we present findings from a unique national survey concerned with vaccine hesitancy conducted with a representative sample of biological and public health scientists who work in research extensive and intensive universities in the US. Specific topics cover the scientific controversy on vaccines, trust in science, reasons for vaccine hesitancy, preferred policy approaches, scientists’ roles in society, science communication, regulation, and ethics. 

Our results provide a deeper appreciation for how the vaccine-related tensions that exist in society play out in the minds of scientists, who are important potential contributors to understanding, responding to, and resolving future crises. This paper begins by reviewing relevant literature and then presents original data and findings before concluding with final insights. 

## 2. Literature

### 2.1. Vaccine Hesitancy: Causes and Consequences 

Despite the success of immunization in preventing diseases during the past century of public health history, vaccine acceptance across the globe is waning [18,19]. The World Health Organization (WHO) listed vaccine hesitancy as one of the key global health threats in 2019 [20]. Vaccine hesitancy, as defined by the WHO, is the reluctance or refusal to vaccinate despite the availability of vaccines. As countries endeavored to develop and distribute vaccines to contain the spread of COVID-19 (Coronavirus), persistent vaccine hesitancy among the public posed serious challenges to the rollout of vaccination programs. In a longitudinal study, Lazarus et al. found that COVID-19 vaccine hesitancy increased from 24.6% in June 2020 to 33.4% in June 2021 in the United States, opposite to the declining trend of the global average [21].

Vaccine hesitancy, as approached in decision-making research, is attributed to multiple causes linked to attitudes and beliefs, cognitive styles, socio-political orientations, educational attainments, availability of vaccines and trust in social authorities [22,23,24,25]. The “3C” model identifies three contributors to vaccine hesitancy: convenience, complacency, and confidence [26]. Convenience is associated with the availability, accessibility, and affordability associated with vaccination. In many cases, these elements are stratified geographically or demographically such that convenience is greater in some communities or groups in society than in others [3]. 

Complacency is associated with the individual-level perceived risks posed by the disease to human health, as well as by the perceived risk of the vaccine. Perceived risk is defined as the subjective appraisal that a hazard will result in an adverse outcome [27]. The lower the perceived risk of the disease and the higher the perceived risk of the vaccine, the higher the potential for vaccine hesitancy [26]. The concept of perceived risk includes cognitive and emotional dimensions [28,29], both of which may operate in decision-making processes [30] during public health emergencies [31,32,33,34]. Determinants of individual-level risk perceptions include disease and vaccine knowledge, voluntariness, visibility of the threat, trust [35], demographic characteristics such as age, gender and ethnicity [36], and socio-cultural institutions [37]. 

Confidence encompasses the level of trust in the safety, efficacy, and delivery system of the vaccine. Institutional trust also plays an important role in building public understanding, mobilizing public cooperation and fostering adherent behaviors [38,39]. Trust in the institutions of government, science, and the healthcare system are important determinants of vaccine hesitancy, such that lower institutional trust generally reduces public confidence in the advice and guidance that the institutions produce [26,38]. In this article, we will draw on aspects of the 3C model to help interpret the scientist’s perspective.

Trust in institutions is undermined by controversy stemming from two sources: from within the scientific community, when experts disagree about the facts and challenge claims of knowledge and from disagreement among the broader set of actors in society, over the validity of technical claims guiding how people should act [40]. Decision-making during crises needs to be fast, even if data quality and accessibility are less than optimal [41]. Under compressed time cycles, such as during the COVID-19 pandemic, the rapid flow of knowledge to policy exposes epistemological uncertainty and the limits of knowledge [42,43]. Public confusion rises and confidence drops when disputes about the technical claims of research fit partisan interests and facilitate disagreement about the implications of research for decision making and individual behavior. 

Trust is also undermined by the second form of controversy generated by competition among information sources, organized disinformation on social media, as well as ambiguous and inconsistent messages from public agencies [44,45,46]. For example, vaccine hesitancy is aided by ineffective and contradictory government communication [47], misleading or bad science [48,49], and misinformation or deliberate disinformation prevalent in social media [44,50,51,52,53]. Research shows that mistrust of science, mainstream media and government are related to susceptibility to misinformation and conspiracy beliefs [54,55], while trust in science is found to be positively related to confidence in vaccination [56].

### 2.2. Overcoming Vaccine Hesitancy: Policy Approaches and Solutions

A key policy question concerns the type of intervention best suited to minimize or reduce vaccine hesitancy. Three general intervention strategies include coercion, incentivization, and persuasion [57,58]. While coercive approaches may be preferred when vaccination is deemed critical for herd immunity and the protection of the whole community, the other strategies rely more on a belief in autonomy and individual health responsibility [59]. 

Vaccine mandates are put in place by institutions that have sufficient coercive power to impose requirements. Evidence indicates that various types of mandates, including employer mandates, can be effective in increasing vaccination rates [60,61]. While these interventions are ideally accompanied by risk-based communication targeted to specific social groups, they are controversial because they do not necessarily balance public welfare concerns related to disease with ethical tensions as they limit individual freedom of choice [59,62]. 

Monetary incentives and non-monetary incentives, such as public recognition, can be used to encourage people to undertake health-related actions [63,64]. Incentive-based interventions (payments and lotteries) were used by different policy communities during the pandemic, yet evidence of effectiveness varies across studies [65,66]. For example, monetary incentives seem to raise vaccination likelihood for those who are reluctant to be vaccinated but not for the unwilling [67]. Meta-analyses find the study effect sizes to be small or non-existent [68,69]. 

Persuasion-oriented strategies, such as nudging, which address decision biases and errors, provide alternative approaches to overcoming vaccine hesitancy. Nudging interventions often take the shape of communication efforts, such as the use of text-based messaging targeting convenience barriers [58] to in-person engagement with healthcare workers targeting confidence barriers [59], which reframe the health risks and options available to elicit desired behaviors [63] without restricting individual choice. The approaches highlight the importance of risk-based decision-making, as noted in the 3C model but extend beyond those concepts to specify decision biases and heuristics that contribute to vaccine hesitancy [70,71]. For example, people generally weigh immediate costs and benefits more heavily than those in the future (present bias), or they are typically more optimistic about their own health risk, judging the risk to be greater for others (optimism bias) [58]. Despite the more targeted approach of persuasive strategies, evidence of their effectiveness is inconclusive [72].

All three strategies rely on a regulatory system that processes scientific evidence from various sources (including pharmaceutical companies as well as from researchers in academia and government). FDA and other federal agencies receive evidence-based advice on vaccinations from the Centers for Disease Control (CDC) and its Advisory Committee on Immunization Practices (ACIP)) to assess the effectiveness and potential risks of health behavior recommendations, diagnostic tools and vaccines. US regulatory agencies, including the Food and Drug Administration (FDA), comprise the “fourth branch” [73] of government, which is responsible for collecting and evaluating scientific evidence supporting rules, standards, and other decisions related to health and safety, including vaccine approvals. Relative to vaccines and diagnostics, the FDA determines “whether there is regulatable risk, as well as the nature and magnitude of the risk and social consequences of regulation” [73] (p. 43). For example, during the COVID-19 pandemic, the FDA served as a “gatekeeper” deciding whether to grant “accelerated approval” or “Emergency Use Authorization” of vaccines and diagnostics under heightened clinical, political and economic pressure [74] without relinquishing standards of quality, safety, and efficacy [75,76].

### 2.3. Scientists’ Roles at the Interface of Science and Society for Vaccine Hesitancy

The success of any intervention is contingent on the risk perception of the public as well as their level of trust in institutions, which would impact their willingness to comply with public health guidance, including vaccination compliance [77,78,79]. Inadequate perceptions of risk and lack of confidence in institutions can both be attributed to ineffective science communication on vaccine hesitancy [80]. 

As a shared responsibility, effective science communication should be reciprocal, transparent, delivered through appropriate platforms, and customized for different groups [80,81]. However, exactly what communication roles do scientists think they should take on? 

It is generally acknowledged that scientists have some responsibility to communicate and engage with the public to transfer knowledge to those outside the scientific enterprise [82,83,84]. Scientists themselves have expressed strong approval of participating in communication efforts, although they are concerned about possible challenges resulting from insufficient institutional support and a lack of communication skills [85]. The WHO highlights the importance of regular and proactive public communication and engagement during public health emergencies to bridge the risk perception gap between the population affected and experts and authorities [86]. Nevertheless, science communication is often criticized, and several studies have shown significant differences of opinion about the quality of science communication during the pandemic [87,88]. According to the Pew Research Center, around half (51%) of Americans believe public health officials did an excellent or good job during the pandemic, while the other half (49%) held opposing attitudes [14].

Determinants of effective communication include the comprehensibility of the message and the identity of the message giver. Surveys of adult Americans indicate that uncertainty highlighted in public communication and political affiliation of the communicator eroded trust in science and science-based policy making during the pandemic [89,90]. A lack of transparency in communication can also undermine trust in institutions [91]. Moreover, scientists are often poorly trained in communication skills, particularly those required during catastrophic events [92,93,94]. Additionally, policy-making is a complex political process in which decision-makers must balance tensions produced by divergent values, ideologies, resources, habits and traditions [95,96,97,98]. While scientific training may be well-suited for structured problems based on explicit and well-founded knowledge, it is less well-suited for complex, uncertain, and value-laden contexts [99,100,101] that require an understanding of resource availability, implementation feasibility, and outcome effectiveness [95,96,97,98]. 

Ineffective science communication can create opportunities for the generation of vaccine controversy during crises, but other factors can make the situation worse. For example, the political nature of decision making can give rise to deliberate suppression or censoring of public or scientific discourse [102], even when there are legitimate concerns over the safety, efficacy, and risks of vaccines [103]. Additionally, controversy is heightened when science lacks methodological rigor or when bias and clinical misconduct is exposed during vaccine research and development [104,105]. Hence, the conduct of science and politics are directly implicated in the trust of these institutions.

Given the brief review of these causes, potential solutions and the role of scientists related to vaccine hesitancy found in the literature, we now turn to the empirical investigation of our primary questions: What do scientists believe are the causes and consequences of vaccine hesitancy? What interventions do they see as best suited to minimize or reduce vaccine hesitancy? What roles do scientists think they have in addressing vaccine hesitancy?

## 3. Materials and Methods

To address the research questions, a cross-sectional survey of 831 members of the Scientists Opinion Panel Survey (SciOPS, which is a science communication survey tool comprising isa panel of academic scientists employed at Research Intensive (R1) universities in the United States. SciOPS aims to collect survey data from randomly selected scientists in different fields to understand general opinions about various topics related to science and the public. Detailed information regarding SciOPS can be found at: https://www.sci-ops.org/, accessed on 1 June 2023). This survey, administered only to biologists and public health academics in the panel, was conducted from 4 April to 1 May 2023. All of the sampled individuals were recruited from an initial sample frame (n = 9649) of current faculty (1) in departments of Biology at Carnegie designated Research Extensive (R1) universities or (2) in accredited schools of Public Health in the United States. Pre-notification electronic messages were delivered on 30 March, informing each sampled individual that a survey invitation would be forthcoming. Email messages containing the formal survey invitation and a hyperlink to the questionnaire were sent on 4 April. Reminder messages were sent on 11 April, 18 April, and 25 April. The survey was closed on 1 May. A total of 316 individuals responded to the online questionnaire for a survey completion rate of 38%. The AAPOR response rate for this survey, which additionally accounts for initial panel recruitment, was 3.3% (The AAPOR response rate for this survey is the Cumulative Response Rate calculated by multiplying the Recruitment Rate for the SciOPS panel (8.8%) with the Completion Rate for this survey (38%) and dividing by 100) [106]. 

The survey questionnaire was administered in English and asked scientists their opinions regarding declining vaccination coverage in the U.S., science and risk communication, and vaccination policies during pandemics. The average time needed to complete the self-administered questionnaire was 10.1 min. All study procedures were approved in advance by the Arizona State University Institutional Review Board. 

Data were weighted to account for probabilities of selection and post-stratified by gender, academic rank, and discipline (Biology vs. Public Health) to represent the population of faculty from which the sample was recruited. The margin of sampling error for survey estimates is +/5 percentage points.

The weighted demographics and background characteristics for the final sample are provided in Table 1. Survey responses were examined using descriptive univariate and bivariate statistics and linear regression models. Comparing survey responses by age, gender, academic field and state politics, we only report statistical test findings with *p*-values lower than 0.05.

We conducted nonresponse analyses to evaluate the degree to which the final sample composition for this survey is (1) representative of the original sample frame of university faculty that was used to recruit SciOPS panel members and also (2) representative of the panelists invited to participate in this survey. Detailed results are reported in Supplementary Material A (Tables S1 and S2). Briefly, females were significantly overrepresented in the final sample of respondents for this survey (*p*-value < 0.005), compared with the gender composition of the original sample frame. We did not observe any additional differences in the demographic composition of the final sample of respondents and the 831 panelists invited to participate in this survey.

## 4. Results

### 4.1. Part I: Controversy, Confusion and Vaccination Behavior

#### 4.1.1. Science Controversy and Public Confusion

**Figure 1:**
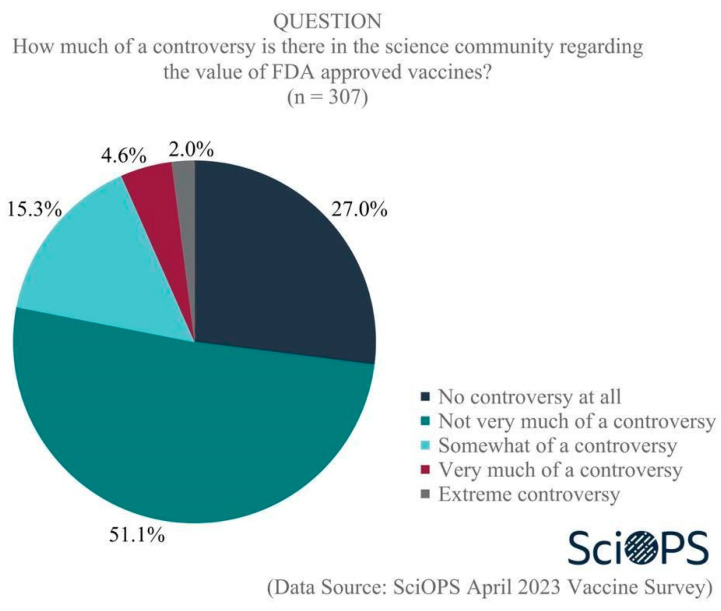
Level of controversy in the science community regarding FDA-approved vaccines.

Our survey findings document the existence of disagreement regarding the level of controversy that exists about vaccines within the scientific community, which can exacerbate the vaccine hesitancy of the population. When asked, “How much of a controversy is there in the science community regarding the value of FDA approved vaccines?”, only one-quarter (27%) of scientists responded that there is no controversy at all (See Figure 1), while about half (51%) indicated that not very much of a controversy exists. Still, approximately one in five (22%) believe that there is somewhat, very much or extreme controversy in the scientific community regarding the value of vaccines. Subgroup analyses revealed that respondents in public health were more likely than biologists to believe there is a controversy (mean difference = 0.3, t = 2.7, *p* < 0.01). 

**Figure 2:**
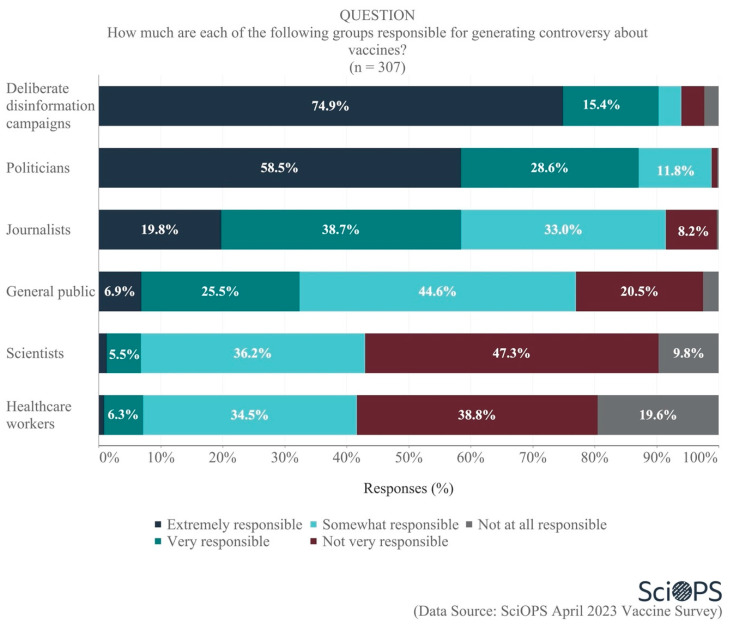
The extent of different groups’ responsibility for generating vaccine controversy.

When asked about the groups who are responsible for generating controversy about vaccines, scientists were very clear in their belief that most of the responsibility for the controversy rests with politicians (87.1% very or extremely responsible) and disinformation campaigns (90.3% very or extremely responsible) (see Figure 2. Some percentages do not sum to 100 due to rounding error.). The second tier of responsible actors included journalists (58.5% very or extremely responsible) and the general public (32.4% very or extremely responsible). Within the general public, some scientists specifically blame those vocal members with limited knowledge about vaccines and poor ability to evaluate information. Lastly, few identified scientists (6.8%) and healthcare workers (7.2%) as responsible for the vaccine controversy. 

Subgroup analyses revealed that female faculty were more likely than male faculty (mean difference = 0.4, t = 3.9, *p* < 0.001), and those located within blue states were more likely than those located within red states (mean difference = 0.3, t = 2.3, *p* < 0.05), to attribute vaccine controversy to deliberate disinformation campaigns. Female faculty were additionally more likely than male faculty (mean difference = 0.3, t = 2.5, *p* < 0.05) to blame the public. Moreover, public health faculty exhibited a higher tendency to attribute vaccine controversy to healthcare workers compared to biologists (mean difference = 0.3, t = 2.5, *p* < 0.05). Meanwhile, female faculty were found to be more inclined than their male counterparts to hold healthcare workers responsible for generating vaccine controversy (mean difference = 0.4, t = 4.1, *p* < 0.001). Increasing age was also associated with (estimate coefficient = −0.15, *p* < 0.05) a greater propensity to blame healthcare workers for generating vaccine controversy.

In open-ended survey items, several respondents additionally identified other groups they believed were also responsible for the controversy. These include science experts at the policy interface, including Dr. Fauci; foreign governments; interest groups; celebrities; religious groups and leaders; family members; social media platforms and influencers; and “Charlatans (people with degrees/credentials: who have never published/participated in the research process who have suddenly declared themselves public health experts”. 

**Figure 3:**
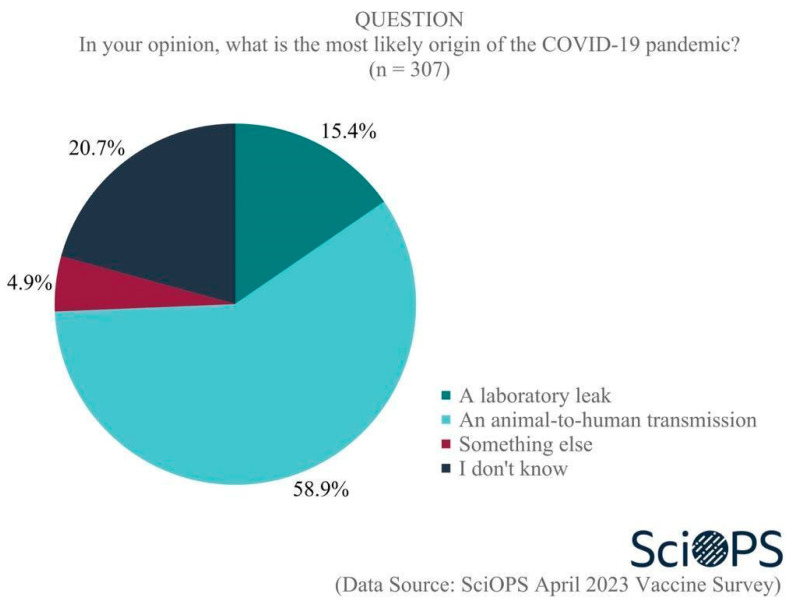
Origin of the COIVD-19 pandemic.

Another serious public controversy concerns the origins of the COVID-19 virus, with scientists in disagreement as well. When asked what they believed the “most likely” origin of the COVID-19 pandemic was, a majority of 58.9% indicated an animal-to-human transmission (See Figure 3. Percentage does not sum to 100 due to rounding error.). A smaller proportion (15.4%) believed a laboratory leak was the source, and one-fifth were uncertain. Among the remaining scientists (4.9%) who reported something else, some believe the answer is too uncertain and complex, while others think it is a combination of a laboratory leak and animal-to-human transmission. Chi-square test results did not show any significant differences among scientists based on age, gender, academic field, and politics of their states with regard to their opinions on the origin of the COVID-19 pandemic.

#### 4.1.2. Causes of Public Confusion about Vaccines and Vaccination

**Figure 4:**
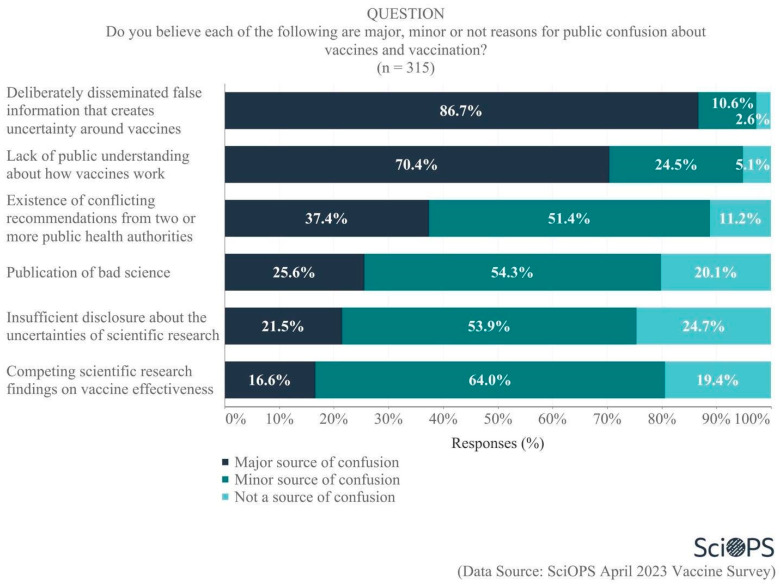
Source of public confusion about vaccines and vaccination.

When asked about sources of public confusion regarding vaccines and vaccinations, most scientists felt that the deliberate dissemination of false information (86.7%) and lack of public understanding (70.4%) were most responsible (See Figure 4. Some percentages do not equal 100 due to rounding error.). Perhaps not surprisingly, more than one-third (37.4%) also believed that conflicting recommendations from public health authorities were a major source of public confusion. Scientists also accepted some responsibility for the public’s confusion, as one-quarter (25.6%) identified the publication of bad science, one-fifth (21.5%) identified insufficient disclosure about the uncertainties of scientific research, and 16.6% cited competing scientific research findings regarding vaccine effectiveness as major sources of confusion. Overall, 43.9% of all scientists acknowledged that bad science, insufficient disclosure of uncertainties, and/or competing findings were a major source of confusion. It is important to note that a relatively large percentage of scientists place some blame on science activities and outputs as a cause for confusion, while only a small minority of scientists believe scientists themselves are responsible for the controversy (see Figure 2). We discuss these different results in the conclusions. 

#### 4.1.3. In What Ways Do Scientists’ Behaviors Reflect Controversy and Confusion?

A small percentage of our sample (14.8%) reports that they had ever “deliberately decided not to get a vaccination when one was recommended”. This finding is slightly lower than vaccine hesitancy levels in recent general population studies, which roughly range between 16 and 22 percent [107,108], but higher than the 12.8% rejection rate for a vaccine with a hypothetical efficacy of 95% [107]. 

For those individuals who decided against vaccination, we followed up with an open-ended question asking the reasons for their decision. The reasons cited for their vaccine hesitancy reflected controversy, risk perception, convenience and personality, also found in the literature [26]. For example, scientists who attribute their vaccine hesitancy behaviors to controversy were skeptical about the vaccine they were recommended. They felt they either lacked sound scientific evidence for the pros and cons of the vaccine to support their vaccination decision, or they thought the efficacy and benefit/risk ratio of the vaccine was too low. When calculating the benefit/risk ratio, one important rationale was the low-risk perception of virus transmission. They found evidence showing that the transmission rate was low. They also believed they were healthy enough to protect themselves from viruses and would have only mild symptoms when infected. One respondent provided a five-point response that summarized several of these points:

1. There was no evidence that the vaccine offered protection for me, as I had recently recovered from the virus it supposedly protected against.
2. The virus was not particularly dangerous.
3. There was no evidence that the vaccine did anything to slow the spread of infection (and some evidence that it somehow increased infection rates).
4. There was ample anecdotal evidence that the vaccine was much more harmful than health authorities admitted.
5. Given 4, there was ample reason to believe that everything else official sources said about the vaccine—and the virus—was misleading and potentially false.

In the open-ended question, others mentioned various personal reasons for having refused a vaccination. These included adverse reactions to vaccines, the belief that vaccination is a low-priority issue for them (e.g., “I did not have time to conveniently get a vaccine—competing priorities”), and/or they had missed the window for a particular vaccination due to inappropriate age (e.g., “I did not get the HPV vaccine because of age and then they changed the age, but I just never got it”,) or health conditions (e.g., “was sick when I had the opportunity”) when they were recommended to receive a vaccine. 

### 4.2. Part II: Policy and Institutions: Trust, Vaccination Strategies and Regulation

#### 4.2.1. US Public Trust in Institutions and Scientists’ Opinions

**Figure 5:**
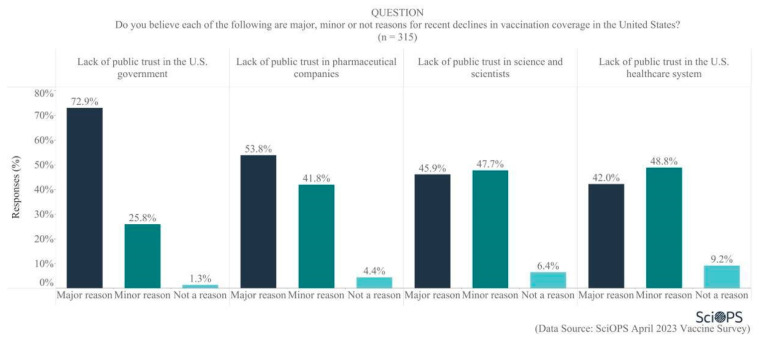
Reasons for recent declines in vaccination coverage in the U.S.

To further explore reasons for declining vaccination coverage in the U.S., our scientist panel was asked about public trust in various types of institutions. Overall, public trust was not thought to be very high, with majorities indicating the belief that lack of public trust in the U.S. government (72.9%) and in pharmaceutical companies (53.8%) were major reasons for declining vaccination coverage (See Figure 5). Scientists were about evenly split regarding whether lack of public trust in science and scientists and in the U.S. healthcare system were major (42.0 and 45.9%, respectively) or minor (47.7% and 48.8%, respectively) reasons for declining coverage. Clearly, scientists are willing to allocate some level of responsibility to science and scientists for declining public trust and its impact on vaccination rates.

Subgroup analyses show that scientists located in blue states were more likely than those located in red states to attribute recent declines in U.S. vaccination coverage to a lack of public trust in the U.S. government (mean difference = 0.1, t = 2.2, *p* < 0.05) and pharmaceutical companies (mean difference = 0.2, t = 2.8, *p* < 0.01). In addition, female faculty were more likely to believe that lack of public trust in pharmaceutical companies is a reason for recent declines in vaccination coverage (mean difference = 0.18, t = 2.7, *p* < 0.01).

#### 4.2.2. Scientists’ Beliefs about Vaccination Strategies and Approaches

**Figure 6:**
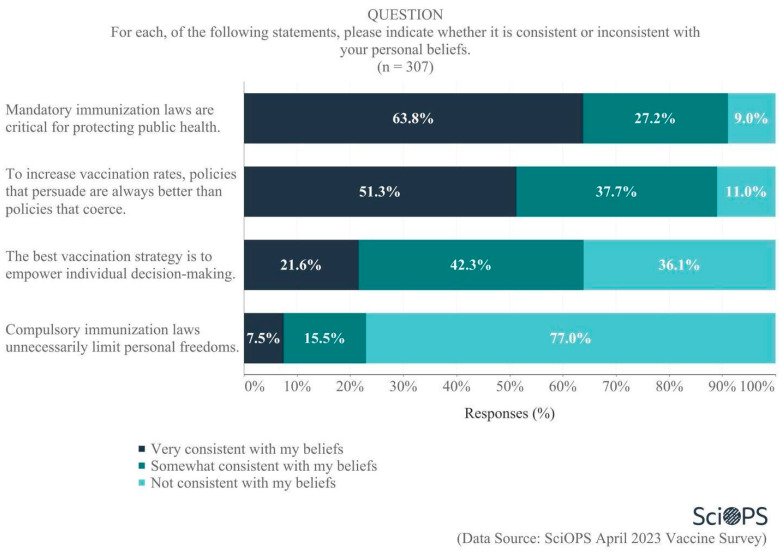
Opinion on policy frames for vaccination.

Scientists were also asked about the extent to which various public policy approaches to vaccine dissemination were or were not consistent with their personal beliefs (Figure 6). Of the four approaches examined, majorities endorsed two that favored coercive approaches:

- Almost two-thirds (63.8%) indicated that the statement “mandatory immunization laws are critical for protecting public health” was consistent with their personal beliefs, and
- More than three-quarters (77%) felt that the statement “compulsory immunization laws unnecessarily limit personal freedoms” was not consistent with their beliefs.

Responses to other policy frames were more nuanced. A majority (51.3%) believed that, in order to increase vaccination rates, “policies that persuade are always better than policies that coerce”. In contrast, there was considerable disagreement regarding the statement “the best vaccination strategy is to empower individual decision-making”, as 42.3% believed this was somewhat consistent with their beliefs, while 36.1% said it was not consistent with their beliefs and 21.6% felt it was very consistent with their beliefs. 

We created an index to measure scientists’ general support for coercive vs. persuasive policy frames for vaccination using their responses to each of the four arguments depicted in Figure 6. The higher the index (range 0–8; alpha = 0.72), the greater a scientist’s willingness to support coercive policies for vaccination (conversely, lower index values represented greater support for persuasive policies). We examined the factors affecting scientists’ general support for these policy frames using a linear regression model. Results show that more senior scientists are slightly less likely than younger scientists to support coercive policy frames (*p* = 0.08). Biologists were also more likely than public health scientists to support coercion (*p* = 0.07). Details of constructing the coercion index (Tables S3 and S4) and linear regression model results (Table S5) are presented in Supplementary Material B.

#### 4.2.3. Risk Benefit Tradeoffs for Expediting FDA Approvals

**Figure 7:**
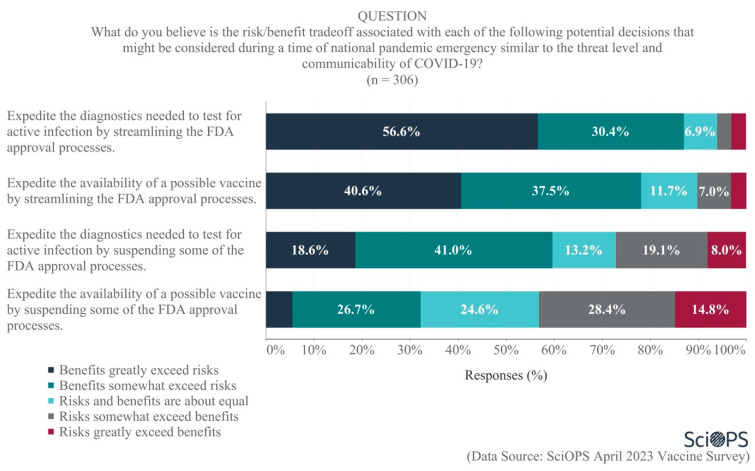
Opinions regarding risks and benefits of expediting FDA approval processes.

Policies that consider risk-benefit tradeoffs could be used as a basis for expediting the development and distribution of new vaccines. Scientists were asked about the risk-benefit tradeoff associated with several potential strategies that “might be considered during a time of national pandemic emergency similar to the threat level and communicability of COVID-19”. The majority felt that the benefits either somewhat or greatly exceeded the risks of expediting the diagnostics needed to test for active infections by either streamlining (87.0%) or suspending (59.6%) some of the FDA approval processes (see Figure 7. Some percentages do not equal 100 due to rounding error.). A majority also believed that the benefits somewhat or greatly exceeded the risks of streamlining FDA approval processes in order to expedite the availability of possible vaccines (78.1%). There was less agreement, though, regarding the risk/benefit ratio of suspending some of the FDA approval processes necessary to expedite vaccine availability. A plurality of scientists believed the risks greatly or somewhat exceeded the benefits of doing so (43.2%), with 32.2% responding that the benefits greatly/somewhat exceeded the risks and about one-quarter (24.6%) believing the risks and benefits were about equal. 

Additional analyses revealed age differences in opinions regarding the diagnostics needed to test for active infections. Specifically, older scientists were less inclined than younger scientists to believe that the benefits of streamlining (estimate coefficient = −0.21, *p* < 0.01) or suspending (estimate coefficient = −0.22, *p* < 0.01) FDA approval processes exceeded the risks of doing so. There were no significant differences among researchers based on other demographic variables examined with regard to these FDA approval questions.

**Figure 8:**
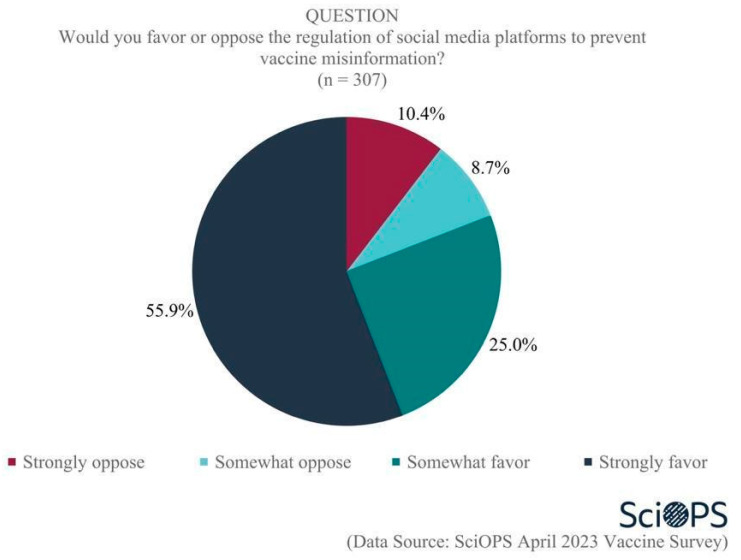
Opinion about regulating social media platforms to prevent vaccine misinformation.

Scientists also strongly favored the idea of preventing vaccine misinformation by regulating social media platforms. As the chart below indicates, 55.9% strongly favored, and 25% somewhat favored some form of social media regulation as a means of preventing the dissemination of vaccine misinformation (Figure 8. Some percentages do not equal 100 due to rounding error.). Female faculty were more likely than male faculty to strongly support the regulation of social media platforms (71.2% and 46.2%, respectively; mean difference = 0.53, t = 4.9, *p* < 0.01). We did not find evidence showing any statistically significant differences among scientists’ attitudes associated with their age, academic field, and state politics.

One additional question was posed to scientists about potential future official actions designed to address vaccine-related disinformation that might be considered: “Recently, the Biden administration halted the implementation of the Department of Homeland Security’s newly created Disinformation Governance Board to coordinate countering misinformation related to homeland security. Do you think there should be a similar government body tasked with countering misinformation and disinformation related to vaccine risks and benefits?” Three-quarters of all respondents (78.5%) supported the idea of establishing a government body to address vaccine-related misinformation, and 21.5% were opposed to the idea. Female faculty were more likely than male faculty to support this idea (mean difference = 0.1, t = 2.6, *p* < 0.01). There were no differences in support by age, academic field, and state politics. 

### 4.3. Part III: Scientists’ Communication Roles and Ethics

#### 4.3.1. Scientists’ Roles

**Figure 9:**
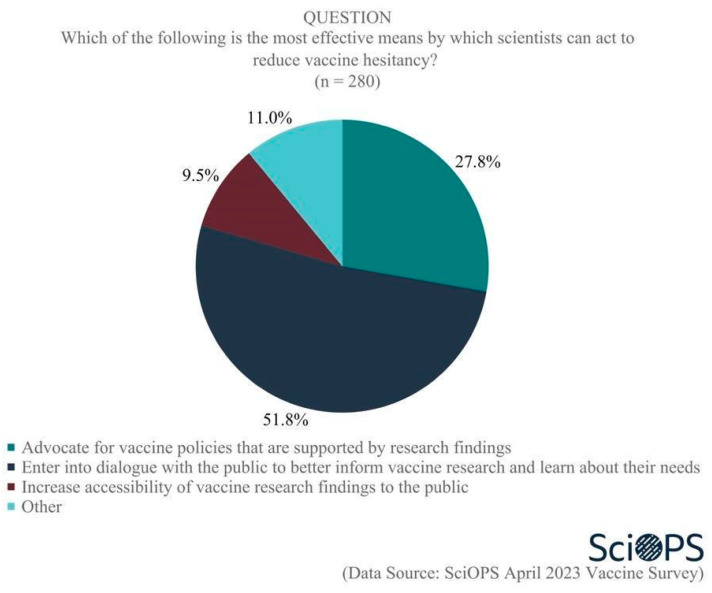
Scientists’ roles in reducing vaccine hesitancy.

A large majority of our sample (90.7%) supported the idea that “scientists have a role in reducing vaccine hesitancy among the public”. When asked the most effective means by which scientists can act to reduce vaccine hesitancy, about half (51.8%) saw their role as through direct dialog with the public to better inform vaccine research and learn about their needs. Another (27.8%) believed the most effective role for scientists was to advocate for vaccine policies that are supported by research findings, while 9.5% believed the most effective means by which scientists could act to reduce vaccine hesitancy was by increasing the accessibility of vaccine research findings to the public. (Figure 9. Percentage does not sum to 100 due to rounding error.). Chi-square test results showed no significant differences among scientists by age, gender, academic field or politics of their states regarding their opinions on the most effective role of scientists in reducing vaccine hesitancy.

Eleven percent volunteered additional thoughts in an open-ended question asking about other potential roles that the science community can play in helping to address vaccine hesitancy. In contrast, a few of the comments were pessimistic about their ability to address vaccine hesitancy, most sought to be constructive. We conducted a modified theme analysis [109] in which the members of the author’s team discussed the thematic method and then separately identified thematic groupings. The authors then agreed on themes and named them with phrases that reflected the content. We identified five general themes: (i) support of the outdated deficit model for science, (ii) engaging the public in nuanced dialogue and two-way communication, (iii) improving knowledge about vaccine hesitancy, (iv) conducting advocacy and activism, especially to address misinformation, and (v) addressing the ethics and equity context of science and society. Representative quotations from survey respondents are presented in the text. A table with other responses for each category is provided in Supplementary Material C.

The first category of open-ended survey responses confirmed the continued existence of the deficit model, which relies on better transfer of information from science to the public, and better education of the public. For example, one respondent wrote: “Educate public about relative risk (of vaccination vs. no vaccination) and how scientists assess it”.

Another group of respondents tended to recognize the limitations of the deficit model, calling for a more nuanced approach to communication that includes greater respect, improved listening, and active dialogue with the public. It was not clear from the comments whether communication was the responsibility of scientists or some other group. One comment provides an example: “Truly listening to and learning from the public. They are not stupid, but we as scientists treat them that way”.

A third category of responses offered ways to increase knowledge about vaccine hesitancy, often in collaboration with the social and behavioral sciences, to develop evidence-based interventions. Some comments related to improving understanding of the root causes of hesitancy. Others focused more on integrating communication expertise. Most respondents recognized that vaccine hesitancy does not necessarily result from scientific misunderstanding but rather relates to the complexities of and context of the communication. For example, one respondent replied: “Collaborate with health communication specialists about how best to communicate benefits and risks of vaccines”.

A fourth category of responses identified the responsibilities of scientists and others to undertake a proactive and even persuasive communication approach. For example, some thought that scientists should be more influential in the network that produces and transmits misinformation about vaccines. Others advocated for a more activist approach to countering misinformation. One scientist said: “I would add that scientists need to self-police their colleagues who publicly promulgate misleading information about vaccine recommendations, benefits, risks etc.”.

Finally, scientists outlined a set of broader responsibilities for making sure science is conducted ethically with transparent standards and that conflicts of interest are addressed. Some offered that the responsibility extends more broadly to ensuring that the system recognizes persistent inequalities in access to healthcare, as well as power differentials in science. As with comments in the other categories, many related to addressing the underlying foundations of public trust in institutions. For example, one respondent answered: “Separate scientific research on vaccines from monetary incentives from both vaccine manufacturers and political organizations”.

#### 4.3.2. Ethics and Vaccine Communication

**Figure 10:**
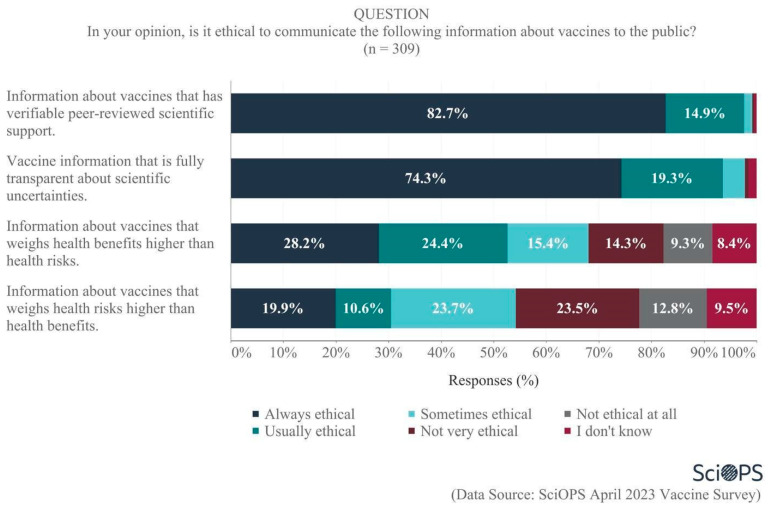
Scientists’ views about the ethics of communicating vaccine information to the public.

Finally, scientists were asked their opinions regarding the ethics of communicating vaccine information with the public. Large majorities felt it was always ethical to communicate information about vaccines that has verifiable peer-reviewed scientific support (82.7%) and that is fully transparent about scientific uncertainties (74.3%) (See Figure 10). When asked to balance the importance of health risks vs. benefits, however, there was less agreement. A majority of 52.6% believed that it was always or usually ethical to communicate information about vaccines that weighs health benefits higher than health risks, whereas only 30.5% felt it was always or usually ethical to communicate information that weighs health risks higher than health benefits. Notably, greater proportions of scientists indicated uncertainty (i.e., do not know responses) regarding the ethics surrounding how much weight to give to health risks vs. benefits when communicating information about vaccines. Clearly, when considering how to communicate beyond the traditional scientific role of reporting peer-reviewed knowledge in a transparent manner, there is less consensus within the scientific community than one might otherwise expect. There were no significant differences among scientists by age, gender, academic field, and politics of their states with regard to their opinions on the ethics of communicating vaccine information with the public. 

## 5. Conclusions

Our goal with this study was to capture scientists’ opinions about vaccine hesitancy in the US along three main lines of inquiry: causes and consequences, policy and regulatory interventions, and scientists’ roles in addressing hesitancy. While in many ways our findings are generally supported by the literature review, they also confirm (1) that scientists, similar to the general population in the US, hold a surprising diversity of perspectives about vaccine hesitancy and (2) that scientists demonstrate a good deal of reflection on the roles science and scientists play both in the generation and potential reduction in vaccine hesitancy. This latter finding is also reflected in other studies of the roles US scientists take as communicators on vaccines, particularly those in the healthcare profession [110,111].

According to the scientists we surveyed, the causes and consequences of vaccine hesitancy can be traced to scientific controversy, public confusion, lack of trust in institutions, and several additional reasons why individuals decide not to vaccinate. A substantial minority of scientists (22%) believe that there is controversy within the science community about vaccine science. However, controversy extends beyond science to society. Scientists place most blame for the broader controversy surrounding vaccines on politicians and disinformation campaigns, but they also hold other segments responsible, including journalists, the public, and to a much lesser extent, scientists and healthcare workers. To put a fine point on controversy, the results show ongoing disagreement among scientists about the origins of the COVID-19 virus.

Most scientists agree that public confusion about vaccines comes from the combination of deliberately disseminated false information and lack of public understanding, but more than two in five (43%) identify the activities of scientists as a reason for confusion (bad science, insufficient disclosure of uncertainties, competing findings). The lack of public trust in societal institutions is another major rationale for declining vaccination rates. Here, scientists see the greatest mistrust of government and pharmaceutical companies, but they also accept that declining trust in science is in part responsible for increases in vaccine hesitancy. Many of these findings are similar to findings from studies of vaccine hesitancy in the general population [26].

The variation in level of responsibility that scientists attribute to controversy and confusion deserves attention. Scientists appear to place primary blame on non-scientist groups for the public controversies about vaccines, but they also believe that practices in the profession of science can cause confusion about vaccines. It is possible that scientists are separating the “politics of controversy”, which they may view as playing out in public, from the influence of science on individual human cognitions and choices. This is potentially a misunderstanding on the part of scientists, who falsely separate science and politics. 

Scientists generally favor coercive strategies for attaining greater vaccine coverage over other strategies that are preferred in the literature, such as persuasion and empowerment. A majority also believes that new regulations are needed to limit deliberate disinformation by social media, and three in four would like to see a new government body tasked with countering misinformation and disinformation related to vaccine risks and benefits. In contrast, scientists think that the regulatory process could be expedited, streamlined, or suspended when the benefits of diagnostics and vaccines exceed the risks. While these two general findings–increasing coercion and reducing regulatory strictness–depend on applying coercive powers of government, they do not accurately reflect the constraints posed by declining trust in government. Moreover, greater discretion in the regulatory process may actually undermine trust in government and science. Hence, it appears that the policy solutions preferred by scientists challenge the realities presented in the US context.

Scientists’ own behaviors also reflect the rationales for vaccine hesitancy found in the literature. While only a relatively small subset (14.8%) admit that they refused to receive vaccination when one was available, the reasons for their decisions cover all elements of the 3C model found in the literature: convenience, complacency, and confidence [26]. 

Yet, what should scientists’ roles be? How do they want to be involved? Somewhat in contrast to their stronger regulatory stances, scientists in our sample expressed the need for true, respectful dialogue, both with the general public and with their colleagues in the social sciences. They suggested several concrete approaches in qualitative responses: (1) listening and genuinely taking the public’s concerns into consideration, (2) actively combating misinformation and disinformation at its source via social media, (3) working with social scientists to better understand the social dynamics that drive hesitancy, and (4) being transparent about uncertainties or gaps in data. Furthermore, our sample overwhelmingly felt that scientists had a responsibility to tackle vaccine hesitancy head-on by either engaging in direct dialogue or in advocacy for evidence-based vaccine policy. 

These findings broadly inform recent science and risk communication research suggesting that addressing controversies such as vaccine hesitancy presents unique challenges that must be approached with a suite of solutions that are narrowly tailored to suit the needs and obstacles present at the community level [112]. On its face, this approach is daunting both from a resource perspective and from a scientist’s role in society perspective. Nevertheless, scientists appear to recognize that strong government or even science-informed regulatory approaches, though desirable, will be increasingly constrained by declining levels of trust in core institutions in society. They sense that a one-size-fits-all communication campaign will likely fall short, especially with a US population that demands more convincing and recognition of personal vaccination decision-making discretion. Additionally, while many express strong responsibility and willingness to be involved, they are acutely aware of their knowledge and training limitations. While, on the one hand, responses belie the persistence of the largely debunked deficit model of science communication in the science community, they also want to collaborate with social scientists and health professions to better address vaccine hesitancy. 

Overall, the findings from this study present a complex portrait of US scientists’ perspective on vaccine hesitancy. We find healthy variation in responses to individual survey questions. Some of this diversity is also found in general population studies. However, we also see that scientists recognize the complexity of the vaccine hesitancy problem and the need to address it through multiple approaches. Although survey findings are based on a representative sample, one key limitation needs to be acknowledged, as our sample is restricted to scientists within two academic disciplines (biology and public health). Although vaccine hesitancy is highly relevant to both disciplines, findings cannot be generalized to scientists outside these two fields. At the same time, it is remarkable that even within this relatively homogeneous sample of experts, the diversity of opinions on most of the topics explored is considerable.

## Figures and Tables

**Table 1:** Descriptive characteristics of survey respondents.

Construct	Variable	(n)	%
Age ^1^	31 to 45	(75)	23.8
	46 to 60	(110)	34.8
	61 to 75	(91)	28.7
	Above 75	(22)	6.9
Gender	Female	(126)	39.8
	Male	(190)	60.2
Field	Biology	(219)	69.4
	Public Health	(97)	30.6
Rank	Full Professor	(132)	41.6
	Associate Professor	(71)	22.4
	Assistant Professor	(68)	21.6
	Non-tenure Track faculty	(46)	14.4
Race ^2^	White	(261)	82.6
	Non-white	(38)	12.0
	Not reported	(17)	5.4
University Region ^3^	Northeast	(73)	23.0
	Midwest	(63)	19.9
	South	(119)	37.8
	West	(61)	19.3
State Politics ^4^	Blue state	(175)	55.3
	Red state	(141)	44.7

^1^ Mean is 56. Standard deviation is 12. Standard error is 0.76. Max is 88. Min is 33. ^2^ Non-white respondents included 1.0% African Americans (n = 3), 9.2% Asians (n = 29), and 1.9% persons reporting mixed races (n = 6). In addition, among our respondents, 5.4% preferred not to self-report their race (n = 17). ^3^ We base our geographic division on the criteria made by U.S. Census Bureau. https://www.cdc.gov/nchs/hus/sources-definitions/geographic-region.htm, accessed on 1 June 2023. ^4^ Measured using the political leanings of the latest elected governor of the states in which the respondents’ universities are located. Data source: https://www.nga.org/governors/, accessed on 1 June 2023.

## Data Availability

The data presented in this study are available on request from Dr. Eric Welch. The data are currently not publicly available during the article review process, but will be made available through the Roper data repository.

## Supplementary Materials

The following supporting information can be downloaded at: https://www.mdpi.com/article/10.3390/vaccines11071208/s1, Supplementary Material A: Non-response Bias Analysis; Table S1: *t*-Test results for demographic differences between survey respondents and original panel recruitment frame; Table S2: *t*-Test results for demographic differences between survey respondents and survey sample; Supplementary Material B: Linear Regression Predicting Scientists’ Support for Coercion vs. Persuasive Policy Frames; Table S3: Question wording and response coding for items used to construct coercion index; Table S4: Frequency and percentage distribution of coercion index: Table S5: Linear regression model results: preference for coercion policy frames; Supplementary Material C: Representative Qualitative Responses.
