# Supplementary material for: How Scientists View Vaccine Hesitancy
Source: Vaccines (Basel). 2023 Jul 6;11(7):1208. doi: 10.3390/vaccines11071208 (PMC10385302; doi:10.3390/vaccines11071208)
Supplement: Supplementary file 1 [file vaccines-11-01208-s001.zip › vaccines-2454012-supplementary.pdf]

Supplementary Materials

The following supporting information will be available for download after publication.

Supplementary Material A. Non-response bias analysis

We conducted two sets of analyses to evaluate potential sources of nonresponse bias in our survey sample. These entail assessments of the degree to which our respondent sample significantly varies across multiple characteristics with (a) the initial sample frame used to recruit scientists to the SciOPS panel, and (b) the sample of panelists selected to participate in this survey.

Table S1 presents t-test comparisons between survey respondents (n=316) and the initial sample frame of Biology and Public Health faculty employed to recruit the SciOPS panel (n=9649). Females were significantly overrepresented in the final survey sample (p-value < 0.005), compared with the gender composition of the initial sample of Biology & Public Health faculty contacted as part of SciOPS panel recruitment (50.6% vs. 40.8%, respectively). There were no observed differences between respondents and the initial sample frame in representation by academic field (i.e., Biology and Public Health). We also did not observe differences by academic rank, although there was a borderline difference in academic rank, with non-tenure track faculty (p-value = 0.092) slightly over-represented in the final sample of respondents (18.4% vs. 14.6% of the initial frame for panel recruitment).

A second set of analyses examined whether there were differences in demographic representation between the sample of 831 respondents invited to participate in this survey and those who agreed to participate (n=316). Table S2 indicates no significant differences by gender, academic field, or faculty rank across these two groups.

Table S1. T-test results for demographic differences between survey respondents and original panel recruitment frame.

**Construct**	**Variable**	**Initial Panel Recruitment sample (%)**	**Respondents (%)**	**Group Differences (%)**	**P-value**
Gender	Female	40.8	50.6	9.9***	0.001
Field	Biology	64.6	64.9	0.3	0.935
	Public Health	35.4	35.1	0.3	0.935
Rank	Full Professor	41.0	42.4	1.4	0.622
	Associate Professor	22.5	20.3	2.2	0.321
	Assistant Professor	21.8	19.0	2.8	0.208
	Non-tenure Track Researcher	14.6	18.4	3.8	0.092

(n)		(9649)	(316)		

* P <0.05, ** p < 0.01, *** p < 0.005

Table S2. T-test results for demographic differences between survey respondents and survey sample.

Construct	Variable	Survey sample (%)	Respondents (%)	Group Differences (%)	P-value
Gender	Female	49.9	50.6	0.7	0.834
Field	Biology	64.6	64.9	0.3	0.936
	Public Health	35.4	35.1	0.3	0.936
Rank	Full Professor	40.1	42.4	2.3	0.475
	Associate Professor	20.6	20.3	0.3	0.903
	Assistant Professor	20.6	19.0	1.6	0.544
	Non-tenure Track Researcher	18.8	18.4	0.4	0.871
(n)		(831)	(316)		

* P <0.05, ** p < 0.01, *** p < 0.005

Supplementary Material B. Linear Regression Predicting Scientists' Support for Coercion vs. Persuasive Policy Frames

To further investigate scientist support for coercive vs. persuasive policy frames, an index was constructed using responses to four survey items (see table below for exact question wording). Response categories for each item were: very consistent with my beliefs, somewhat consistent with my beliefs, not consistent with my beliefs. Table S3 presents response coding for purposes of index construction.

Table S3. Question wording and response coding for items used to construct coercion index.

For each of the following statements, please indicate whether it is consistent or inconsistent with your personal beliefs.	Very consistent with my beliefs	Somewhat consistent with my beliefs	Not consistent with my beliefs
Mandatory immunization laws are critical for protecting public health.	2	1	0
To increase vaccination rates, policies that persuade are always better than policies that coerce.	0	1	2
The best vaccination strategy is to empower individual decision making.	0	1	2
Compulsory immunization laws unnecessarily limit personal freedoms.	0	1	2

Table S4. Frequency and percentage distribution of coercion index.

Coercion index (n=306)	0	1	2	3	4	5	6	7	8
(n)	13	12	17	23	35	61	74	51	20
%	4.2	3.9	5.6	7.5	11.4	20.0	24.2	16.7	6.5

The coercion index ranged from 0-8, with higher values representing greater support for coercive vaccine policies and lower values representing greater support for persuasive policy frames. Coefficient alpha for the index was 0.72. The mean value of the coercion index was 5.0 (SD = 2.0). The frequency and percentage distribution for the coercion index variable is reported in Table S4. Subgroup analyses do not show any evidence suggesting there are significant differences among scientists in relation to demographic variables (age, gender, academic field, and state politics) with regard to their overall general preference for the coercion policy frame.

The Coercion index was examined as the dependent variable in an OLS regression model, which is presented in Table S5. Findings indicate that age is borderline inversely associated with support for coercive policy frames, as younger scientists were somewhat less likely than older scientists to support coercion policies ($p = 0.08$). Biologists were also more likely than public health scientists to support coercion at a borderline level of significance ($p = 0.07$). In this model, gender, academic rank and state level politics were not associated with support for coercive or persuasive policy frames.

Table S5. Linear regression model results: preference for coercion policy frames.

	Estimate	Standard Error	P-value
(Intercept)			
Age	-0.02	0.01	0.08*
Gender			
Female	0.18	0.24	0.46
Field			
Biologist	0.57	0.31	0.07*
Rank			
Assistant Professor	-0.43	0.46	0.36
Associate Professor	0.24	0.35	0.49
Non-tenure track faculty	-0.21	0.34	0.53
State politics			
Red state	-0.20	0.26	0.42
Observations	287		
R-square	0.08		
Note: * p<.1<br>Reference groups: male, public health professionals, full professor, blue state.			

Supplementary Material C: Representative Qualitative Responses

Category 1: Support of the outdated deficit model for science

- "Explain the science in terms that are understandable to the public, and how knowledge about vaccines is developed, including the implications for protecting the health of the public through mitigation of spread of an infectious disease."
- "Present ongoing information in clear, easy to understand language about work being done on vaccines…focus on literacy and numeracy level."
- "Educate public about relative risk (of vaccination vs no vaccination) and how scientists assess it."

Category 2: Engaging the public in nuanced dialogue and two-way communication

- "Truly listening to and learning from the public. They aren't stupid, but we as scientists treat them that way."
- "[Apply] the learning cycle, listening to people's prior knowledge first and having known people in the community as advocates are probably the way forward"
- "Listen to the reasons behind the hesitancy."
- "Discuss with the public especially in informal settings but be mindful in the ways we communicate about stuff as communicating to the public in a less than ideal manner can increase distrust."

Category 3: Improving knowledge about vaccine hesitancy

- "Research the nature and causes of vaccine hesitancy and design evidence-based interventions to counter it."
- "Understand reasons for hesitancy and address them."
- "Vaccine research scientists need to take a back seat to social scientists and others who have more training and expertise in human behavior, psychology, and other social and political structures that shape vaccine hesitancy."
- "[Work] with social scientists and communications experts how to reach hesitant audiences…"
- "Collaborate with health communication specialists about how best to communicate benefits and risks of vaccines."
- "Conduct community-based participatory evidence-based intervention trials to increase community-science dialogue in planning and implementing research interventions…."

Category 4: Conducting advocacy and activism, especially to address misinformation

- "I would add that scientists need to self-police their colleagues who publicly promulgate misleading information about vaccine recommendations, benefits, risks etc."
- "Reframe how vaccines are talked about [and] inundate news and social media with news and stories…from…mothers, kids, influencers, regular folk. [Convince people] to want a vaccine without making it seem like they changed their mind"
- "Increase the visibility of scientists and improve accessibility of research language in publications in order to start rebutting the highly visible and outspoken charlatans who advocate for misinformation."
- "Understand the channels that push out misinformation and their motivations/profits from it. Use established methods for countering this information."
- "Become influential on the networks that mischaracterize or lie about vaccine efficacy."

Category 5: Addressing the ethics and equity context of science and society

- "Vaccine hesitancy has become a social movement that is linked to a range of attitudes scientists cannot address very well, except by ensuring the larger field 'science' is as ethical as possible."
- "Advocate for thresholds to determine empirical support -- e.g., established conventions for risk/benefit ratio, replicated findings done by multiple independent research groups, etc."
- "Science, unfortunately, has suppressed leading, and highly credited, individuals with specific concerns about mRNA-based methods, not vaccines in general."
- "Separate scientific research on vaccines from monetary incentives from both vaccine manufacturers and political organizations."
- "Advocate and support robust public health and social structures [so that] they care for people, [with] less inequalities, and thereby engender greater trust."
